# Englerin A Selectively Induces Necrosis in Human Renal Cancer Cells

**DOI:** 10.1371/journal.pone.0048032

**Published:** 2012-10-22

**Authors:** Florian J. Sulzmaier, Zhenwu Li, Mika L. Nakashige, David M. Fash, William J. Chain, Joe W. Ramos

**Affiliations:** 1 Cancer Biology Program, University of Hawaii Cancer Center, University of Hawaii at Manoa, Honolulu, Hawaii, United States of America; 2 Department of Chemistry, University of Hawaii at Manoa, Honolulu, Hawaii, United States of America; 3 Department of Molecular Biosciences and Bioengineering, University of Hawaii at Manoa, Honolulu, Hawaii, United States of America; The Ohio State University, United States of America

## Abstract

The number of renal cancers has increased over the last ten years and patient survival in advanced stages remains very poor. Therefore, new therapeutic approaches for renal cancer are essential. Englerin A is a natural product with a very potent and selective cytotoxicity against renal cancer cells. This makes it a promising drug candidate that may improve current treatment standards for patients with renal cancers in all stages. However, little is known about englerin A's mode of action in targeting specifically renal cancer cells. Our study is the first to investigate the biological mechanism of englerin A action in detail. We report that englerin A is specific for renal tumor cells and does not affect normal kidney cells. We find that englerin A treatment induces necrotic cell death in renal cancer cells but not in normal kidney cells. We further show that autophagic and pyroptotic proteins are unaffected by the compound and that necrotic signaling in these cells coincided with production of reactive oxygen species and calcium influx into the cytoplasm. As the first study to analyze the biological effects of englerin A, our work provides an important basis for the evaluation and validation of the compound's use as an anti-tumor drug. It also provides a context in which to identify the specific target or targets of englerin A in renal cancer cells.

## Introduction

Kidney cancer is one of the most common malignancies in the U.S with an estimated 60,920 new cases and 13,120 deaths in 2011. About 85% of all kidney cancers are classified as renal-cell carcinomas, a malignancy arising from the renal epithelium [1], [2]. The primary therapy for patients with renal-cell carcinoma is surgical excision. However, approximately 25% of the patients show signs of local invasion or metastasis making a complete excision difficult [2]. Once the disease is in an advanced stage, surgery alone is not sufficient and the 5-year patient survival drops from 70% to under 20% [1], [3]. Historically, the state of the art treatment for patients with renal cell carcinoma has been immunomodulatory therapy with interferon-α or interleukin-2 [4]. However, recent years have seen an increase in the use of more targeted approaches to treat advanced stage renal cancers. The discovery of the von Hippel-Lindau (VHL) tumor suppressor gene lead to the development of receptor tyrosine kinase-based therapies targeting the VEGF or TGF-α signaling pathway [5], [6]. VHL regulates angiogenesis and a loss of this gene in cancer cells results in increased production of growth factors like VEGF [7]. Alternatively, receptor tyrosine kinase inhibitors like Sorafenib that block signaling through affected pathways are now approved or in clinical trials for the treatment of advanced renal cancers [8]. However, these drugs are not applicable for all patients with advanced renal cancers and severe side effects have been reported in some cases [2], [9].

Englerin A is a guaiane sesquiterpene that showed intriguing specificity as inhibitor of renal cancer cell growth [10]. The natural product was isolated from the bark of *Phyllanthus engleri*, a plant native to Tanzania and Zimbabwe. Englerin A was screened for specific cytotoxic activity against a panel of cancer cell lines (NCI 60-cell panel). In this screen, the compound showed renal cancer specific GI_50_ values that were up to 1000fold lower than in other cancer cell lines. GI_50_ values determined were as low as 11 nM for certain renal cancer cell lines [10], [11]. Englerin A not only showed extraordinary specificity for renal cancer cells, in some cases its potency was even higher then state of the art treatments like Sorafenib [10], [12]. After its initial description in 2009, labs around the world have described synthetic strategies to make the natural product [13], [14], [15], [16], [17], as well as growing amounts of structure-activity relationship data [11], [18], [19], [20], [21], [22]. Despite its high impact, literature on englerin A is still limited and published articles mainly deal with the synthesis of the compound. We now report for the first time a mechanism by which englerin A acts on renal cancer cells to inhibit cell growth. Our results show that englerin A specifically induces necrotic cell death in renal cancer cell lines, but does not affect the viability of a glioblastoma cancer cell line or normal kidney cells. Our study provides further insight into the biological activity of englerin A.

## Materials and Methods

### Reagents

Englerin A was synthesized in the laboratory of Dr. William J. Chain according to the protocol previously published [12]. Staurosporine was purchased from EMD Chemicals (Merck, Darmstadt, Germany), ionomycin and chloroquine diphosphate was purchased from Sigma-Aldrich (Sigma-Aldrich Corp., St. Louis, MO).

### Cell Lines and Cell Culture

Human renal cell carcinoma lines A-498 and UO-31, as well as the human glioblastoma cell line SF-295 were obtained from the DCTD Tumor Repository of the National Cancer Institute, Frederick, Maryland. Cells were cultured in RPMI-1640 (Mediatech Inc., Manassas, VA) containing 10% fetal bovine serum (FBS, Life Technologies, Grand Island, NY), 1% MEM nonessential amino acids (NEAA, Mediatech) and 1% penicillin-streptomycin (PenStrep, Mediatech). HEK293 cells were purchased from ATCC (Rockville, MD) and maintained in DMEM (Mediatech) supplemented with 10% FBS, 1% NEAA and 1% PenStrep. Renal proximal tubule cells (RPTC) were purchased from Lifeline Cell Technology (Frederick, MD). Cells were cultured in RenaLife complete medium (Lifeline Cell Technology). All cells were cultured at 37°C and 5% CO_2_ in a humidified incubator.

### Cell Viability Assay

Cells were plated in 96-well plates in 90 μl of RPMI without phenol red and without antibiotics, supplemented with 10% FBS and 2mM L-glutamine. Cell were seeded at a density of 5,000 cells per well. Cells were allowed to anchor down for 60min at 37 °C and 5 % CO2 in a humidified atmosphere. After 60 min, 10 μl of englerin A working solution or an equivalent volume of DMSO diluted in RPMI medium was added. An englerin A stock solution was prepared by dissolving the compound in DMSO at a concentration of 10 mM. All further englerin A working solutions were prepared by diluting this stock solution with RPMI medium to the desired final concentration as indicated. The volume of englerin A stock solution or DMSO carrier control therefore never exceeded 0.1 % of the final volume. After adding the compound, cells were incubated for 48h. Cell viability was determined using a Cell Proliferation Assay (XTT) according to the manufacturer's protocol (Roche Diagnostics, Indianapolis, IN).

### Microscopy

Brightfield micrographs were taken using a Zeiss Axiovert200M microscope with a 40x objective. Acquired images were analyzed using AxioVision software. Scale bars represent 20 μm.

### Annexin V/PI assay

Cells were treated with either 1 μM englerin A, carrier DMSO or 5 μM staurosporine for the indicated amount of time (1h or 3h). After incubation, cells were trypsinized and stained for extracellular phosphatidyl serine expression using FITC-tagged Annexin V and propidium iodide (PI) as co-stain to test cell membrane integrity (BD Biosciences, San Jose, CA). Dyes were used according to the manufacturer's protocol. Stained cells were analyzed using a FACScan flow cytometer (BD Biosciences) and CellQuest Pro analyzing software.

### Cell lysis and immunoblotting

Cell lysates were prepared using MLB lysis buffer (1% NP-40, 25 mM HEPES-KOH (pH 7.5), 150 mM NaCl, 0.25% sodium deoxycholate, 10% glycerol, 10 mM MgCl_2_, 1mM EDTA and protease/phosphatase inhibitors). Cell lysates were resolved using SDS-PAGE, followed by immunoblotting. Protein expression was detected with specific primary antibodies against PARP, caspase 3 and GAPDH (Cell Signaling Technology, Danvers, MA), as well as caspase 1 (EMD Millipore, Billerica, MA), Beclin-1 (Epitomics, Burlingame, CA) and LC-3 (Novus Biologicals, Littleton, CO). Binding of primary antibodies was detected using IRDye 680 goat anti-mouse and IRDye 800 goat anti-rabbit secondary antibodies. Bands were visualized using an Odyssey Infrared Imaging System (Li-Cor Biosciences, Lincoln, NE).

### Caspase 3 activity assay

Caspase 3 activity in cells treated with englerin A, staurosporine or DMSO carrier control was analyzed using a Caspase 3 Activity Assay Kit (Roche Diagnostics). After incubation with the compound cells were lysed and cell lysates were assayed according to the manufacturer's protocol.

### ROS detection assay

Cells treated with englerin A, staurosporine or carrier control DMSO were tested for their production of reactive oxygen and nitrogen species using the Total ROS Detection Kit (Enzo Lifesciences, Farmingdale, NY). Cells were treated according to the manufacturer's protocol. The relative change in reactive oxygen or reactive nitrogen species was measured using a FACScan flow cytometer (BD Biosciences) and CellQuest Pro analyzing software.

### Intracellular Ca^2+^ assay

Intracellular calcium concentration was measured after cells were treated with englerin A, carrier DMSO or ionomycin positive control. After 30min incubation with the compound 2 μM Fluo-3 AM (Life Technologies) was added for subsequent 30min incubation. After incubation, cells were trypsinized and washed with 1x PBS. Fluo-3 binding to Ca^2+^ ions was measured through an increased fluorescence emission of the dye at 520 nm upon excitation at 485 nm. Cells were analyzed using a FACScan flow cytometer (BD Biosciences) and CellQuest Pro analyzing software.

## Results

### Chemically synthesized englerin A reduces viability of renal cancer cell lines

**Figure 1:**
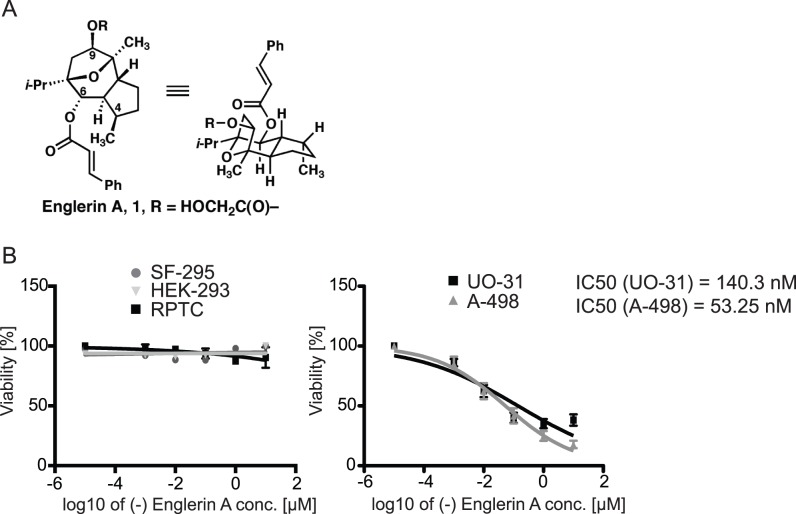
Englerin A selectively reduces cell viability in renal cancer cells. (A) Chemical Structure of englerin A. (B) Glioblastoma (SF-295), normal immortalized kidney cells (HEK-293), renal proximal tubule cells (RPTC) and renal cancer cells (UO-31, A-498) were incubated with the indicated concentration of englerin A for 48 h. Cell viability was analyzed using an XTT Cell Proliferation Assay. Results are shown in % viability compared to a cell sample treated with the carrier DMSO. Values shown represent the mean ± SEM of all experiments (n≥6). IC50 values were calculated with Prism 5 using a non-linear regression fit (log(inhibitor) vs. normalized response – variable slope).

Englerin A has been described to have potent activity in inhibiting renal cancer cell growth [10]. Recent publications describe methods for a synthetic production of englerin A without the need of isolating the natural product [13], [14], [16], [17]. All englerin A in our study (chemical structure see Fig. 1*A*) has been synthesized following the protocol described in our recent article by Li and colleagues [12]. In order to evaluate the potency of the synthetic englerin A we screened its cytotoxic effects on two human renal cancer cell lines (UO-31 and A-498) that have been described before to be sensitive to the natural product. As control cell line we used a human glioblastoma cell line (SF-295), previously reported to be unresponsive to englerin A [10]. Furthermore we analyzed the effects of englerin A treatment on the viability of an immortalized kidney cell line derived from normal human embryonic kidney cells (HEK293) and normal human renal epithelial cells (renal proximal tubule cells, RPTC). Viability was determined by measuring the metabolic activity of the cells.

We found that englerin A reduced the viability of renal cancer cells while it had no cytotoxic effects on the glioblastoma control cell line (Fig. 1*B*). Interestingly, the compound did not affect the viability of HEK293 cells either and only changed cell viability in RPTCs at very high concentrations (Fig. 1*B*, *left*). IC_50_ values were determined as 140.3 nM for UO-31 cells and 53.25 nM for A-498 cells. The IC_50_ for RPTCs was approximately seven magnitudes higher and was calculated as 2.53 M.

### Englerin A causes morphological changes different from staurosporine-induced apoptosis

To exclude englerin A effects on cell proliferation that would have accounted for the observed decrease in metabolic activity we analyzed cell morphology after treatment with englerin A using a brightfield microscope. Morphological changes also allowed us to distinguish between apoptotic and necrotic cell death. For this analysis we furthermore compared the cell morphology in response to incubation with staurosporine, a known inducer of apoptosis [23].

**Figure 2:**
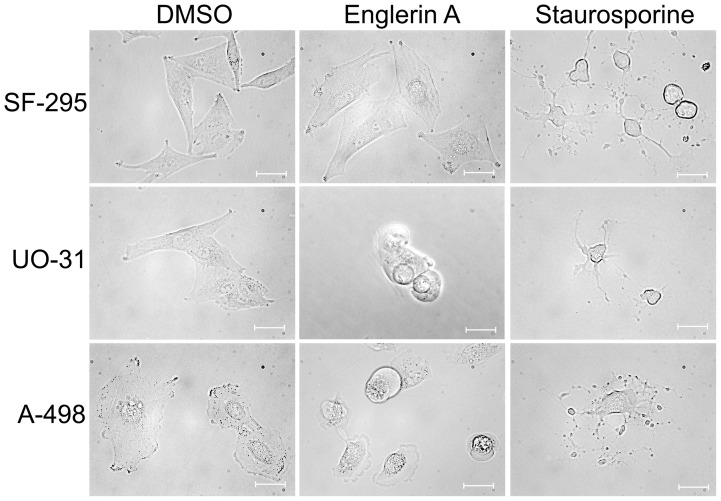
Englerin A induces cell death morphologically distinct from staurosporine induced apoptosis. Micrographs show the morphology of cells treated with englerin A or staurosporine, a known inducer of apoptosis. Cells were treated with either 1 μM englerin A or carrier DMSO for 60 min, or 1 μM staurosporine for 5 h. Pictures were taken using a Zeiss Axiovert200 M microscope with a 40× phase objective. For every treatment, 5–10 random fields of vision were acquired. The experiment was repeated three times, micrographs shown are representative of the average cell morphology upon treatment. Scale bars represent 20 μm.

We found that englerin A treatment of renal cancer cells resulted in an obvious change in cell morphology pointing to cell death (Fig. 2). No differences in cell shape or structure could be observed in glioblastoma cells SF-295 treated with the compound. Renal cancer cells treated with englerin A lost filopodia extensions, eventually reaching a round, symmetrical structure, before fully detaching from the matrix. Staurosporine induced apoptosis in both glioblastoma and renal cancer cells. Apoptotic cell death was characterized by shrinkage of the cells and the formation of apoptotic bodies surrounding the dying cell. Although the treatment with englerin A caused a relative decrease in cell volume, we could not observe the formation of clear apoptotic bodies. Both staurosporine and englerin A caused the renal cancer cells to die, but in morphologically distinct ways.

### Englerin A treatment results in a loss of membrane integrity, but not in the up-regulation of external phosphatidyl serine indicative of early apoptotic stages

**Figure 3:**
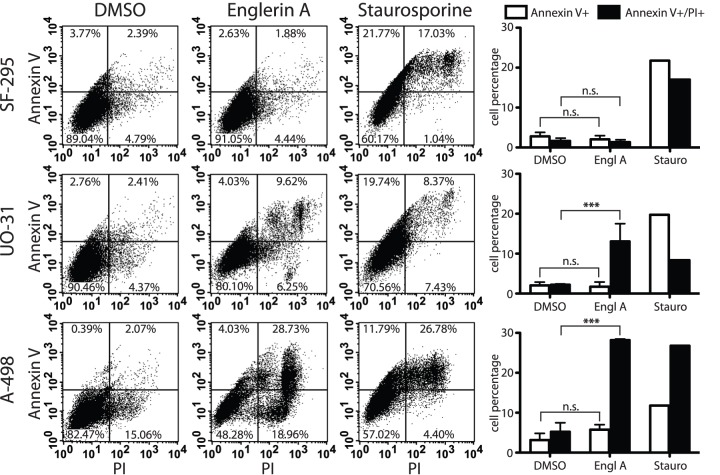
Englerin A does not lead to up-regulation of extracellular phosphatidyl serine. Cells were treated with either 1 μM englerin A or carrier DMSO for 60 min, or 5 μM staurosporine for 3 h. After incubation, cells were trypsinized and stained for extracellular phosphatidyl serine expression using FITC-tagged Annexin V and propidium iodide (PI) as co-stain to test cell membrane integrity. Shown is a result representative of three independent experimental repeats. Quantifications and statistics of all data are depicted as bar graphs and show the distribution of cells testing positive for Annexin V binding (early apoptotic stages) or Annexin V binding and propidium iodide uptake (late apoptotic stages/necrotic death). Values shown are mean ± SEM (n = 3), statistically significant differences are marked with asterisks (*** p<0.001), n.s. = not significant.

The morphologically different outcome in cells treated with the apoptosis-inducer staurosporine led us to analyze if renal cancer cells die through necrotic signaling processes rather than apoptosis when incubated with englerin A. However, direct measures of necrosis are scarce and the most common way to confirm necrotic cell death is by excluding that a cell dies through apoptosis [24]. Early apoptotic stages are characterized by an increase of phosphatidyl serine (PS) on the extracellular side of the cell membrane followed by the loss of membrane integrity in the late apoptotic stages [25]. We used FITC-labeled Annexin V and propidium iodide to analyze these two parameters by flow cytometry, which is a common approach to determine if cell death is apoptotic or necrotic [26], [27]. We quantified the cell subpopulations corresponding to early apoptotic and late apoptotic/necrotic stages by measuring the percentage of cells expressing extracellular PS with or without the loss of membrane integrity (Fig. 3).

As expected, the control cell line SF-295 did not show a significant increase in apoptotic cell populations when treated with englerin A or the DMSO carrier control. Staurosporine treatment caused apoptotic cell death in all cell lines accompanied by an increase in both early and late apoptotic cell populations. Renal cancer cells A-498 were the most sensitive, showing the highest percentage (27%) of late apoptotic/dead cells. SF-295 and UO-31 cell lines showed elevated populations in the early apoptotic stages of about 12–20%. A clear increase in early apoptotic cells was also visible after 1h of treatment with staurosporine (Fig. S1).

Interestingly, englerin A treatment did not affect the cell populations in the same way. We found no significant change of early apoptotic cell populations (Annexin V single positive) in either UO-31 or A-498 cells after treatment with englerin A. The treatment caused the cells to lose membrane integrity early, leading to a double positive staining of the cells. While the percentage of cells in the early apoptotic sector did not increase, we observed a statistically significant increase to approximately 10–15% dead cells in UO-31 cell samples and a double positive population of up to 27% in A-498 cells after 1 h of treatment (Fig. 3, bar graphs). Even at a later time-point the number of single positive, early apoptotic cells did not increase in the englerin A treatments (Fig. S1). DMSO treatment of both renal cancer cell lines (UO-31 and A-498) did not induce a significant up-regulation of either single positive (Annexin V) or double positive (Annexin V and PI) populations.

### Englerin A induced cell death is independent from PARP cleavage and Caspase 3 activity

**Figure 4:**
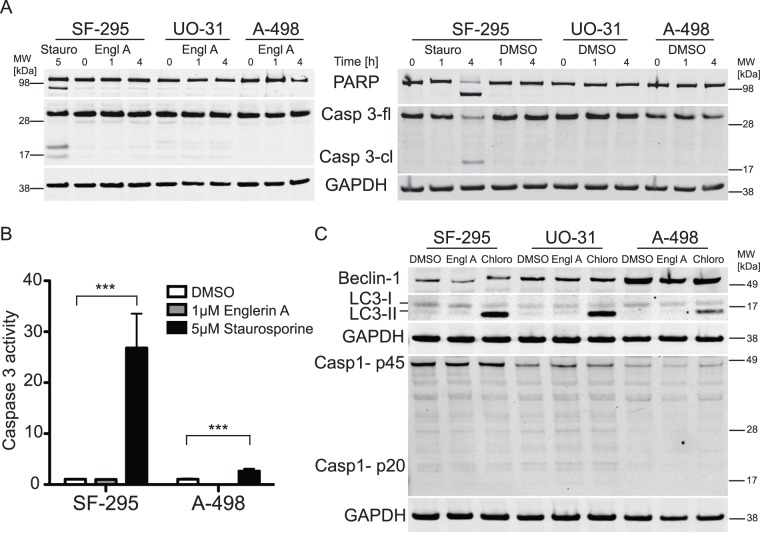
Englerin A does not induce cleavage of caspase 3, PARP, caspase 1 or the autophagic markers LC-3 and Beclin-1. Cells were treated with either 1 μM englerin A, carrier DMSO or 5 μM staurosporine for the indicated amount of time. (A) After the incubation, cells were lysed and lysates were analyzed by immunoblotting for PARP cleavage or full-length and cleaved caspase 3 (Casp3-fl, Casp3-cl). Equal protein loading was confirmed by probing for GAPDH. Full-length and cleaved bands are indicated. The experiment was repeated three times. (B) Alternatively, after incubation cells were lysed and caspase 3 activity was tested using a caspase 3 activity assay kit. Values shown are means ± SEM (n = 6), statistically significant differences are marked with asterisks (*** p<0.001). (C) Cells were treated with either 1 μM englerin A, carrier DMSO for 60min or 50 μM chloroquine diphosphate (Chloro) for 18 h. After the incubation, cells were lysed and lysates were analyzed by immunoblotting for Beclin-1, LC3-I/II and caspase 1 cleavage (proenzyme p45 and cleaved active subunit p20). Equal protein loading was confirmed by probing for GAPDH. All membranes were analyzed using IRDye secondary antibodies and a Licor Odyssey system. Membranes shown are from representative experiments.

Apoptotic cell death is in part mediated by effector caspases like caspase 3 that cleave and activate downstream targets like poly(ADP-ribose) polymerase (PARP). PARP is thought to aid in apoptotic signaling by depleting the cells energy resources [28]. Cleavage and activation of these proteins is therefore a marker for an activated apoptotic signaling cascade. To further confirm that renal cancer cells treated with englerin A do not die through apoptosis we tested the cleavage of PARP (Fig. 4*A*) and the cleavage and activity of caspase 3 (Fig. 4*A and B*). As a control, treatment of SF-295 cells with staurosporine for 4 or 5 h resulted in cleaved protein bands at 17 and 19 kDa indicative of the active protein fragments. Similarly, staurosporine treatment of SF-295 cells caused the cleavage of PARP as indicated by the 89kDa cleaved fragment. We found that englerin A did not affect the cleavage of these two proteins in SF-295 or the renal cancer cells (UO-31 and A-498). We could not detect bands for cleaved caspase 3 or PARP when treating the cell lines with englerin A for 1 or 4 hours (Fig. 4*A*). Control treatments of all cell lines with the carrier DMSO for the same amount of time did not result in detectable cleavage of caspase 3 or PARP (Fig. 4*A,* right panel).

To confirm this result we analyzed caspase 3 activity with an ELISA based assay that measures enzymatic activity directly through the cleavage of a caspase 3 substrate. As expected, staurosporine treatment resulted in an increase of caspase 3 activity in both the glioblastoma control cell line and the renal cancer cell line A-498. Englerin A treatment did not lead to any significant increases in enzyme activity indicative of caspase 3 activation (Fig. 4*B*).

### Englerin A does not induce caspase 1 cleavage

Pyroptosis is a mode of cell death caused by inflammation pathways. Signaling is independent from the apoptosis-related effector caspases 3 and 7, but involves the release of active interleukin-1β mediated by caspase 1 [29], [30]. Here we measured caspase 1 cleavage as an indicator of pyroptotic cell death. We followed the dynamics of caspase 1 activation by detecting the levels of the 45 kDa pro-enzyme and the 20 kDa reduced caspase 1 isoform after treatment with englerin A (Fig. 4*C*). Interestingly, we found that caspase 1 is activated to a low level in both tested types of cell lines, with differing expression levels that are highest in SF-295 cells and lowest in A-498 cells. We detected slight bands for cleaved caspase 1 isoforms in samples of SF-295 glioblastoma cells and UO-31 renal cancer cells. However, treatment with englerin A did not significantly increase the levels of caspase 1 cleavage indicative of pyroptotic signaling. Band intensities for the cleaved caspase 1 fragment are similarly low. Levels of p45 pro-enzyme barely change upon englerin A treatment (Fig. 4*C*)

### Englerin A does not induce autophagy

Autophagy is a cellular process in which cytoplasmic material is degraded with the help of lysosomes. The mechanism per se is a recycling pathway that is generally associated with cell survival. However, there are reports of cells undergoing a mode of cell death in which cells up-regulate autophagic signaling (even though autophagy is not the cause of cell death in this scenario). The result is called autophagic cell death [31], [32]. The activation of autophagy can be analyzed by following the processing of the autophagic marker LC3 and its conversion from the LC3 I isoform to the LC3 II form that is accompanied by a change in molecular weight [31]. Another earlier marker for autophagy is Beclin-1, which is up-regulated upon induction of autophagy and triggers the formation of autophagosomes [33], [34].

We determined if englerin A treatment induced autophagy in the experimental cell lines by following changes in LC3 and Beclin-1 (Fig. 4*C*). As a positive control for the detection of the LC3 II isoform we treated all cell lines with 50 μM chloroquine (Chloro) for 18 h, a substance shown to arrest autophagy at the autophagosomal stage resulting in increased levels of LC3 II [35], [36]. Treatment with chloroquine resulted in a strong increase of LC3 II levels in all tested cells. The LC3 I isoform was detectable in all cell lines under all treatment conditions. However, treatment with englerin A or the carrier DMSO did not result in a significant up-regulation of LC3 II. Even though we were able to detect faint bands for this LC3 isoforms in SF-295 and UO-31 cells, the level if LC3 II did not significantly increase upon englerin A treatment (Fig. 4*C*).

Beclin-1 levels could be detected in all experimental cell lines. Lowest levels were found in SF-295 cells, highest levels in A-498 cells. Treatment with englerin A or chloroquine did not result in differences in Beclin-1 levels in either glioblastoma or renal cancer cells. Englerin A did not lead to a significant up-regulation of Beclin-1 expression levels (Fig. 4*C*).

### Englerin A causes the production of reactive oxygen species

**Figure 5:**
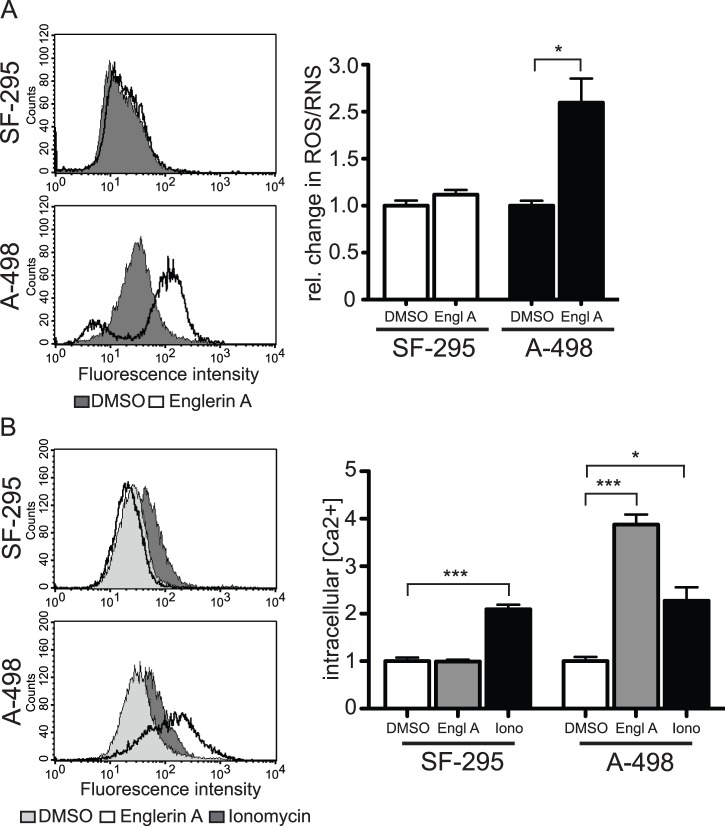
Englerin A induces production of reactive oxygen species and increased concentration of intracellular Ca^2+^. (A) Cells were treated with either 1 μM englerin A or carrier DMSO for 60 min. The relative change in reactive oxygen (ROS) or reactive nitrogen species (RNS) compared to cells treated with the carrier DMSO was measured using the Total ROS detection kit. Histograms show fluorescence intensities in a representative experiment (left panel). Quantified relative changes in ROS/RNS shown (right panel) are means ± SEM (n = 5), statistically significant differences are marked with asterisks (* p<0.05). (B) Cells were treated with either 1 μM englerin A or carrier DMSO for 60 min, or 10 μM ionomycin for 50 min. Fluo-3 binding to Ca^2+^ ions was measured through an increased fluorescence emission of the dye at 520 nm upon excitation at 485 nm. Histograms show fluorescence intensities in a representative experiment (left panel). Quantified relative changes in intracellular calcium ions shown (right panel) are means ± SEM (n = 3), statistically significant differences are marked with asterisks (* p<0.05, *** p<0.001).

Oxidative stress induced by excessive production of reactive oxygen species (ROS) is a known factor causing necrotic cell death [24]. We therefore sought to analyze if englerin A caused an increase of intracellular ROS. We treated SF-295 and A-498 cells with the compound and measured the content of total reactive oxygen and nitrogen species (Fig. 5*A*).

In our study, englerin A did not affect the relative amount of ROS in SF-295 cells when compared to a treatment with the carrier control DMSO. However, A-498 cells reacted strongly to englerin A by producing ROS concentrations significantly higher than in the control cells. The total amount of reactive species was about 2.5-fold higher when A-498 cells were treated with englerin A.

### Englerin A induces an increase in intracellular Ca^2+^ concentration

Calcium has been shown to regulate necrotic signaling. Excessive influx of extracellular Ca^2+^ into the cells can stimulate both the production of reactive oxygen species and necrotic cell death [24]. We measured the effects of englerin A on intracellular Ca^2+^ concentrations using the calcium indicator Fluo-3. This dye allowed us to quantify the amount of intracellular calcium ions after treatment with englerin A (Fig. 5*B*). The relative Ca^2+^ concentration did not change when SF-295 cells were incubated with englerin A. Ionomycin, a known inducer of Ca^2+^ influx into the cell, doubled the measured ions [37]. Treatment of A-498 renal cancer cells with englerin A significantly increased intracellular calcium to an even higher extent then ionomycin. While ionomycin doubled the amount of intracellular calcium, englerin A resulted in 4-fold higher concentrations.

## Discussion

The increased incidence of renal tumors around the world presents a serious problem [1], [38]. For patients with renal tumors in advanced stages, effective chemotherapeutics are scarce and commonly used drugs like Sunitinib have been associated with serious side effects [9]. The search for new therapeutics therefore has to be focused on treatments that are not only effective in fighting the tumors, but also specific enough to not harm non-tumor cells and to avoid adverse side effects. The natural product englerin A is a compound that potentially fits these criteria. Its high selectivity and potency against renal cancer cells put it in the focus of many research groups. However, little is known about its mode of action, besides targeting these cells with a high specificity [10]. In order to fully evaluate englerin A's use as a therapeutic agent and to anticipate possible side effects it is necessary to understand the mechanism by which the compound affects renal cancer cells.

Here we show for the first time how englerin A kills renal cancer cells. Importantly, we also report that englerin A is in fact specific for cancerous renal cells and does not affect normal kidney cells. As to the mode of action, we found that englerin A induces a necrotic form of cell death in the sensitive cells. Apoptotic markers like phosphatidyl serine externalization, effector caspase activation or PARP cleavage are not up-regulated after treatment with englerin A. Plasma membrane permeabilization happens quickly and cells don't form apoptotic bodies. At the same time autophagy levels are not affected by the treatment and the compound does not seem to induce pyroptosis-like processes. However, the mode of cell death includes an increased production of reactive oxygen species and rising levels of intracellular calcium ions either as part of the necrotic signaling or a result thereof.

The potency of a compound is an important measure of its qualification as a drug. Half maximal inhibitory concentrations (IC_50_) in the nanomolar range or lower are desirable. At the same time a low IC_50_ increases the likelihood of a good selectivity of the drug. With IC_50_ values in the lower nanomolar range (140.3 nM for UO-31 cells and 53.25 nM for A-498 cells) englerin A's has a potency that makes it a suitable drug candidate. Tests with non-renal or non-cancer cell types also proved its extraordinary selectivity. While significantly reducing the viability of renal cancer cells, englerin A does not assert cytotoxic effects on glioblastoma cells (SF-295) or immortalized human kidney cells (HEK-293) in concentrations of more then two magnitudes higher then the IC_50_ for renal cancer cells. Englerin A did not affect viability of normal human kidney cells either as the IC_50_ values tested in renal proximal tubule cells show. The compound's IC_50_ values were approximately 7 magnitudes higher in this cell type. Englerin A therefore shows both potency and selectivity for renal cancer cells.

Over the last decades, it became clear that the pathways leading to cell death are very diverse and a simple classification into only two categories, apoptosis and necrosis, based on one test is not sufficient. The Nomenclature Committee on Cell Death therefore proposed a system that helps separating the different ways of cell death by their morphological features and the cellular signaling pathways involved. Their guidelines help classifying modes of cell death by analyzing a set of different parameters [24], [39]. Using this classification system we were able to characterize the mode of death induced by englerin A as part of the necrotic types of cell death. Brightfield pictures of cells treated with the compound led us to believe early on that the mechanism was different from typical apoptotic inducers like staurosporine. The lack of apoptotic body formation and the quick loss of membrane integrity, which started as early as 20–30 min after the start of the treatment with englerin A, pointed towards necrotic rather than apoptotic signaling. However, to be certain we analyzed the changes in cell morphology and signaling in more detail to confirm our initial observations. Unfortunately there is no test measuring distinct markers for necrotic signaling [24]. In order to confirm necrosis it is therefore common practice to rather test for the absence of apoptotic markers. A lack of phosphatidyl serine up-regulation on the plasma membrane, the failure to detect activation of effector caspases and missing cleavage of PARP eliminated a possible involvement of apoptotic signaling. Among the non-apoptotic death pathways we were able to exclude autophagic cell death due to the lack of LC3 processing and the absence of changes in Beclin-1 levels, leaving our initial hypothesis of a necrotic cell death confirmed. The nonexistence of caspase 3 activation made pyroptosis a likely candidate for the mode of cell death. Recent publications connected this atypical cell death modality to a multi-protein complex called the inflammasome, which includes caspase 1 and leads to the production of interleukin 1β, a process relevant for certain inflammatory reactions [30], [39]. However, caspase 1 was not activated by englerin A treatment eliminating this candidate as well.

Typical inducers of necrotic signaling include an overproduction of reactive oxygen species (ROS) and an uncontrolled influx of calcium ions into the cytoplasmic space [24]. Interestingly, we were able to detect both events after treatment of renal cancer cells with englerin A. The increase of total ROS was easily detectable after only 30 min, as well as a significantly higher intracellular calcium concentration. Both parameters are therefore likely to be involved in the cell death induced by englerin A. However, the exact signaling pathways leading to the production of ROS and the influx of calcium in this scenario are not known. Further investigation is necessary to clarify the exact involvement of these molecules in the necrotic process.

Differential expression of proteins regulating the influx of ions into the cell and the production of ROS might play a role in causing englerin A's selectivity for renal cancer cells. Factors that could contribute to malfunctions in these areas are imbalance of channel proteins, a change in membrane composition or a disturbance in the mitochondrial machinery [24]. Englerin A could develop a selectivity for cells that already have a predisposition for a disturbance of these processes due to differential expression of the proteins involved. It has been shown that a variety of ion channels and transmembrane transporters are overexpressed in tumor tissues, including channels for K^+^, Na^+^ or Ca^2+^ ions [40]. In human renal cell carcinomas in particular two potassium ion channels of the ether-a-go-go (EAG) family were reported to be up-regulated [41]. An additional possible target is CXCR4, a C-X-C chemokine receptor that has been reported by multiple groups to be up-regulated in renal cancers [42]. The receptor transduces signals across the plasma membrane by increasing the intracellular Ca^2+^ ion concentration [43]. Deregulation of this receptor could also contribute to an abnormal Ca^2+^ influx and cause cellular damage.

**Figure 6:**
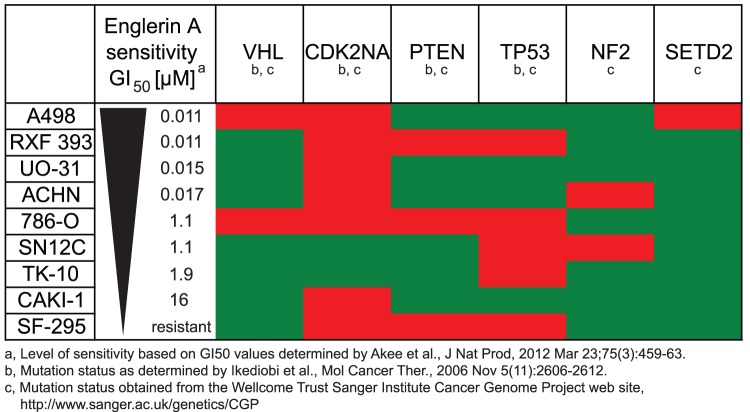
Englerin A sensitivity does not correlate with any known mutations in kidney cancer cell lines. Renal cell carcinoma cell lines from the NCI-60 cell panel and a glioblastoma cell line (SF-295) were arranged by decreasing sensitivity to the englerin A natural product as determined by Akee and colleagues. [11] The known mutation status in the cell lines as characterized by Ikediobi and colleagues [44] and as obtained from the Wellcome Trust Sanger Institute Cancer Genome Project web site (http://www.sanger.ac.uk/genetics/CGP) are shown in red (mutated) or green (wild-type).

Renal cancer cells harbor a set of known mutations that distinguish them from normal kidney cells [44]. The Von Hippel-Lindau (VHL) tumor suppressor gene is the most prominent gene that is frequently inactivated in renal cancers and mutations in this gene have been associated with the development of sporadic clear cell renal carcinomas [45]. We analyzed if the mutation status of genes commonly mutated in renal cancer cells correlates with the sensitivity of these cells to englerin A. The somatic mutations we compared affected the genes *VHL*, *CDK2NA*, *PTEN, TP53*, *NF2* and *SETD2* (Fig. 6). A correlation of this kind might give further insight into why englerin A is so selective in killing renal cancer cells. We correlated the sensitivity of all renal cancer cell lines from the NCI-60 panel and the SF-295 glioblastoma control cell line to the cells' known mutations [11], [44]. We found no functional correlation between englerin A sensitivity and mutation status of the analyzed genes. Mutations in these genes are therefore most likely not linked to englerin A sensitivity.

We conclude that englerin A is an effective cytotoxic agent that reduces the viability of renal cancer cells at a low concentration, but at the same time does not harm other cell types including normal kidney cells. In renal cancer cells, englerin A activates necrosis with coincident production of ROS and calcium influx. Necrotic cell death can contribute to inflammatory reactions, and this is a potential concern. However, englerin A does not induce inflammasome formation, potentially reducing concerns about undesirable inflammatory side effects. Our study is an important first step in evaluating englerin A's possible use as an anti-cancer therapeutic. Even though further research is necessary to fully understand the molecular targets of englerin A in renal cancer cells, our work provides a valuable basis for a better understanding of englerin A's biological actions. Englerin A remains one of the most promising drug candidates in development for curing kidney cancer without the severe side effects of other treatments.

## Supporting Information

Figure S1 **Englerin A induced cell death follows a distinct time course and differs morphologically from staurosporine induced apoptosis.** Cells were treated with either 1 μM englerin A or 5 μM staurosporine for 1 h or 3 h. After incubation, cells were trypsinized and stained for extracellular phosphatidyl serine expression using FITC-tagged Annexin V and propidium iodide (PI) as co-stain to test cell membrane integrity. Shown is a representative result of three independent experimental repeats. Dot plots show cells testing positive for Annexin V binding (early apoptotic stages) in the upper left quadrant and cells positive for Annexin V binding and propidium iodide uptake (late apoptotic stages/necrotic death) in the upper right quadrant. Numbers shown represent quadrant percentages related to total number of cells.
(EPS)
